# Supplementary material for: Interactions of Opuntia ficus-indica with Dactylopius coccus and D. opuntiae (Hemiptera: Dactylopiidae) through the Study of Their Volatile Compounds
Source: Plants (Basel). 2024 Mar 27;13(7):963. doi: 10.3390/plants13070963 (PMC11013929; doi:10.3390/plants13070963)
Supplement: Supplementary file 1 [file plants-13-00963-s001.zip › plants-2915631-supplementary.pdf]

Interactions of *Opuntia ficus-indica* and *Dactylopius coccus* or *D. opuntiae* (Hemiptera: Dactylopiidae) through the study of its volatile compounds

Rodríguez-Leyva E.[1], García-Pascual E.[2], González-Chávez M. M. [3*], Méndez-Gallegos S. de J.[2*], Morales-Rueda J. A.[4], Posadas-Hurtado J. C.[3], Bravo-Vinaja A.[2], Franco-Vega A.[3]

1 Colegio de Postgraduados, Posgrado en Fitosanidad, Entomología y Acarología, Campus Montecillo, CP 56264 Texcoco, Estado de México, México.
2 Colegio de Postgraduados, Posgrado en Innovación en Manejo de Recursos Naturales, Campus San Luis Potosí, CP 78622 Salinas de Hidalgo, San Luis Potosí, México.
3 Universidad Autónoma de San Luis Potosí, Facultad de Ciencias Químicas. Dr. Manuel Nava Martínez #6, Zona Universitaria, C.P.78210, San Luis Potosí, San Luis Potosí, México; gcmm@uaslp.mx
4 Viscoelabs. Material Research Center. Av. Real de Lomas No. 350 local 113, Lomas 4ta sección, CP 78216 San Luis Potosí, México.
* Correspondence: M.M.G.-C. gcmm@uaslp.mx; Tel.: +52 4448262300 Ext.6471; S. J. M.-G. jmendez@colpos.mx Tel.: +52 4969630240 Ext. 4030

SUPPORTING INFORMATION

List of Contents

Table S1. Yields of essential oils of *Dactylopius* species and *Opuntia* varieties. 2
Table S2. SIMPER analysis of *Dactylopius* species 2
Table S3. SIMPER analysis of *Opuntia* varieties. 6
Table S4. SIMPER analysis of *Opuntia ficus-indica* (OFI) Esmeralda and OFI Esmeralda infested by *Dactylopius coccus*. 9
Table S5. SIMPER analysis of OFI Rojo Pelón and OFI Rojo Pelón infested by *Dactylopius opuntiae* 12

Figure S1. Comparison of volatilomes of the OFI Rojo Pelón uninfested and infested by *D. opuntiae* using Venn diagram. 15
Figure S2. Comparison of volatilomes of the OFI Esmeralda uninfested and infested by *D. coccus* using Venn diagram. 15
Figure S3. *Dactylopius opuntiae* compounds grouped. The data are presented as the mean of the peak area of each compound (grouped by type) and the range of the data. 16
Figure S4. *Dactylopius coccus* compounds grouped. The data are presented as the mean of the peak area of each compound (grouped by type) and the range of the data. 17
Figure S5. OFI Rojo Pelón compounds grouped. The data are presented as the mean of the peak area of each compound (grouped by type) and the range of the data. 17
Figure S6. OFI Esmeralda compounds grouped. The data are presented as the mean of the peak area of each compound (grouped by type) and the range of the data. 18

Table S1. Yields of essential oils of *Dactylopius* species and *Opuntia* varieties.

Essential oils	Amount obtained (mg)	Yield (%)
Dactylopius coccus	106.7	0.11
Dactylopius opuntiae	102.0	0.10
Opuntia ficusindica Esmeralda	127.1	0.01
Opuntia ficusindica Esmeralda infested by *D. coccus*	126.6	0.01
Opuntia ficusindica Rojo Pelón	138.6	0.01
Opuntia ficusindica Rojo Pelón infested by *D. opuntiae*	201.9	0.02

Table S2. SIMPER analysis of *Dactylopius* species

Compounds	Averange dissimilarity	Contribution %	Cumulative %
Ethyl octanoate	0.4484	2.128	2.128
p-Cymene	0.4484	2.128	4.255
Benzylmethylether	0.4484	2.128	6.383
Benzophenone	0.4484	2.128	8.511
Butanedioic acid	0.4484	2.128	10.64
Glycolic acid	0.4484	2.128	12.77
Ethyl Benzoate	0.4484	2.128	14.89
Ethyl decanoate	0.4484	2.128	17.02
ß-Ionone	0.4484	2.128	19.15
2-Ethylhexanol	0.4484	2.128	21.28
Ethyl hexadecanoate	0.4484	2.128	23.4
Octanal	0.4484	2.128	25.53
Octadecane	0.4484	2.128	27.66
Ethyl nonanoate	0.4484	2.128	29.79
Decanal	0.4484	2.128	31.91
Dodecanal	0.4484	2.128	34.04
2,4-dimethylnonanoic acid	0.4484	2.128	36.17
Phenol	0.4484	2.128	38.3
Ethyl octadecanoate	0.4484	2.128	40.43
a-Ionona	0.4484	2.128	42.55
2,4-dimethylhexanoic acid	0.4484	2.128	44.68
Ethyl tetradecanoate	0.4484	2.128	46.81
Ethyl tetradecanoate	0.4484	2.128	48.94
Nonanedioic acid	0.4484	2.128	51.06
2-Decenoic acid	0.4484	2.128	53.19
1-Tridecene	0.4484	2.128	55.32
1-Tridecanol	0.4484	2.128	57.45
2,3,4-Trimethylpentanoic acid	0.4484	2.128	59.57
2-Nonadecanone	0.4484	2.128	61.7
Tridecanoic acid	0.4484	2.128	63.83
1-Octadecanol	0.4484	2.128	65.96
2-methylhexanoic acid	0.4484	2.128	68.09
(Z)-9-Hexadecenoic acid	0.4484	2.128	70.21
2,6-dimethylheptanoic acid	0.4484	2.128	72.34
Undecanoic acid	0.4484	2.128	74.47
Heptadecanoic acid	0.4484	2.128	76.6
Heneicosane	0.4484	2.128	78.72
Ethyl Dodecanoate	0.4484	2.128	80.85
Hexanoic acid	0.4484	2.128	82.98

Heptadecanal	0.4484	2.128	85.11
Heptanal	0.4484	2.128	87.23
Hexanal	0.4484	2.128	89.36
cis-5-Dodecenoic acid	0.4484	2.128	91.49
1-Tetradecanol	0.4484	2.128	93.62
1-Hexadecanol	0.4484	2.128	95.74
Dodecanoic acid	0.4484	2.128	97.87
2-Ethylhexanoic acid	0.4484	2.128	100
Tetradecanoic acid	1.86E-09	8.82E-09	100
Decanoic acid	1.33E-09	6.32E-09	100
a-Hexylcinnamaldehyde	1.12E-09	5.30E-09	100
Lactic acid	9.86E-10	4.68E-09	100
1-Dodecanol	8.81E-10	4.18E-09	100
Hexadecanoic acid	7.50E-10	3.56E-09	100
(Z,Z)-9,12-Octadecadienoic acid	6.12E-10	2.90E-09	100
Nonanal	4.66E-10	2.21E-09	100
(Z)-9-Octadecenoic acid	3.38E-10	1.61E-09	100
Dehydroabietic acid	3.05E-10	1.45E-09	100
Nonanoic acid	2.61E-10	1.24E-09	100
Benzoic acid	2.09E-10	9.91E-10	100
Octadecanoic acid	1.86E-10	8.82E-10	100
Hexadecane	1.44E-10	6.84E-10	100
(Z)-4-tert-butylcyclohexyl acetate	1.42E-10	6.74E-10	100
Hexylsalicylate	1.00E-10	4.76E-10	100
Heptanoic acid	9.19E-11	4.36E-10	100
Octanoic acid	6.27E-11	2.97E-10	100
(Z)-9-Tetradecenoic acid	4.18E-11	1.98E-10	100
p-Hydroxybenzoic acid	3.97E-11	1.88E-10	100
p-Hydroxybenzoic acid	3.97E-11	1.88E-10	100
3-Ethoxy-4-hydroxybenzaldehyde	0	0	100
Heptadecane	0	0	100
4-Hydroxy-3-methoxybenzaldehyde	0	0	100
3-(4-(tert-butyl)phenyl-2-methylpropanal	0	0	100
Octacosanol	0	0	100
Nonanaldimethylacetal	0	0	100
Salicylic acid	0	0	100
3,7,11,15-Tetramethyl-2-hexadecenol	0	0	100
Phenylacetaldehyde	0	0	100
p-Vinylguaiacol	0	0	100
2,5-Dimethoxy benzenemethanol acetate	0	0	100
Benzeneacetic acid	0	0	100
4-Methylvaleric acid	0	0	100
5,5-Dimethyl-3-oxo-1-cyclohexene-1-carboxaldehyde	0	0	100
1-Octanol	0	0	100
Diethyl acetal hexanal	0	0	100
Benzylalcohol	0	0	100
Benzaldehyde	0	0	100
Heptanol	0	0	100
Hexanol	0	0	100

ß-Sitosterol	0	0	100
Glutaric acid	0	0	100
Neophytadiene	0	0	100
Dihydroactinidiolide	0	0	100
Methyl octadecanoate	0	0	100
Methyl 2-methoxy benzoate	0	0	100
2-Hexenoic acid	0	0	100
a-Isomethylionone	0	0	100
ß-Damascenone	0	0	100
28-Nor-17ß(H)-hopane	0	0	100
Nerol	0	0	100
1,2-Dihydroxy-4-methylpentane	0	0	100
2-Methoxybenzoic acid	0	0	100
(Z)-2-Hexen-1-ol	0	0	100
Geraniol	0	0	100
Linalool	0	0	100
a-Terpineol	0	0	100
ß-Linalool	0	0	100
15-Methylhexadecanoic acid	0	0	100
Guaiacol	0	0	100
Nonanol	0	0	100
Glycerol	0	0	100
Gliceric acid	0	0	100
1-Methyl-1(4-methyl-3-cyclohexenyl)ethanol	0	0	100
Isododecanol	0	0	100
Methyl benzoate	0	0	100
1,5,5-Trimethyl-3-methylene cyclohexene	0	0	100
trans-Linalool oxide	0	0	100
Linalool oxide	0	0	100
Limonene	0	0	100
Benzyl salicylate	0	0	100
1,2-Dihydro-1,1,6-trimethyl naphthalene	0	0	100
10,18,Bisnorabieta-8,11,13.triene	0	0	100
1-(1-cyclohexen-1-yl)(-1-1-Butenone)	0	0	100
Isopropyl tetradecanoate	0	0	100
Nonadecane	0	0	100
Eicosane	0	0	100
2-(1-Hydroxybut-2-enylidene)cyclohexanone	0	0	100
4-Oxoisophorone	0	0	100
Pentadecanoic acid	0	0	100
Heptanoic acid	0	0	100
Phenylacetone	0	0	100
Isophorone	0	0	100
Nonanedioic acid	0	0	100
Acetophenone	0	0	100
2,2,6-Trimethylcyclohexanone	0	0	100
Ethyl salycilate	0	0	100
5-Hexen-2-one	0	0	100
1,2-Dimethoxybenzene	0	0	100
Benzyl Benzoate	0	0	100

2,3-Dihydro-2,2,4,6-tetramethylbenzofuran	0	0	100
Methyl benzoate	0	0	100
Lactic acid	0	0	100
3-Hydroxy-2-methylpyran-4-one	0	0	100
3-Ethyl-4-methyl-1H-pyrrole-2,5-dione	0	0	100
3-Isobutyl-2-methoxypyrazine	0	0	100
Ethyl 2-(5-methyl-5-vinyltetrahydrofuran-2-yl)propan-2-yl carbonate	0	0	100
12-Methyltridecanoic acid	0	0	100
2-Nonenoic acid	0	0	100
2-Methoxy-3-isopropylpyrazine	0	0	100
2-Isopropyl-3-metoxypirazina	0	0	100
Octadecanal	0	0	100
Docosane	0	0	100
Methyl tetradecanoate	0	0	100
Methyl salicylate	0	0	100
2-Methyl-4-pentenoic acid	0	0	100
4-Oxopentanoic acid	0	0	100
3-Methyl-2-pentenoic acid	0	0	100
Heptanoic acid	0	0	100
Phenylacetone	0	0	100
Isophorone	0	0	100
Nonanedioic acid	0	0	100
Acetophenone	0	0	100
2,2,6-Trimethylcyclohexanone	0	0	100
Ethyl salycilate	0	0	100
5-Hexen-2-one	0	0	100
1,2-Dimethoxybenzene	0	0	100
Benzyl Benzoate	0	0	100
2,3-Dihydro-2,2,4,6-tetramethylbenzofuran	0	0	100
Methyl benzoate	0	0	100
Lactic acid	0	0	100
3-Hydroxy-2-methylpyran-4-one	0	0	100
3-Ethyl-4-methyl-1H-pyrrole-2,5-dione	0	0	100
3-Isobutyl-2-methoxypyrazine	0	0	100
Ethyl 2-(5-methyl-5-vinyltetrahydrofuran-2-yl)propan-2-yl carbonate	0	0	100
12-Methyltridecanoic acid	0	0	100
2-Nonenoic acid	0	0	100
2-Methoxy-3-isopropylpyrazine	0	0	100
2-Isopropyl-3-metoxypirazina	0	0	100
Octadecanal	0	0	100
Docosane	0	0	100

Methyl tetradecanoate	0	0	100
Methyl salicylate	0	0	100
2-Methyl-4-pentenoic acid	0	0	100
4-Oxopentanoic acid	0	0	100
3-Methyl-2-pentenoic acid	0	0	100
3-Isobutyl-2-methoxypyrazine	0	0	100
Ethyl 2-(5-methyl-5-vinyltetrahydrofuran-2-yl)propan-2-yl carbonate	0	0	100
12-Methyltridecanoic acid	0	0	100
2-Nonenoic acid	0	0	100
2-Methoxy-3-isopropylpyrazine	0	0	100
2-Isopropyl-3-metoxypirazina	0	0	100
Octadecanal	0	0	100
Docosane	0	0	100
Methyl tetradecanoate	0	0	100
Methyl salicylate	0	0	100
2-Methyl-4-pentenoic acid	0	0	100
4-Oxopentanoic acid	0	0	100
3-Methyl-2-pentenoic acid	0	0	100

Table S3. SIMPER analysis of *Opuntia ficus-indica* varieties.

Compounds	Averange dissimilarity	Contribution %	Cumulative %
4-Oxoisophorone	0.5005	1.088	1.088
β-Damascenone	0.5005	1.088	2.175
β-Ionone	0.5005	1.088	3.263
Nerol	0.5005	1.088	4.351
Acetophenone	0.5005	1.088	5.438
2-Nonenoic acid	0.5005	1.088	6.526
Glycerol	0.5005	1.088	7.614
3,7,11,15-Tetramethyl-2-hexadecenol	0.5005	1.088	8.702
Decanal	0.4978	1.082	9.783
Methyl benzoate	0.4978	1.082	10.87
Benzyl Benzoate	0.4978	1.082	11.95
Linalool	0.4978	1.082	13.03
Hexadecane	0.4878	1.06	14.09
Phenol	0.4878	1.06	15.15
Nonanaldimethylacetal	0.4878	1.06	16.21
Diethyl acetal hexanal	0.4878	1.06	17.27
Hexylsalicylate	0.4878	1.06	18.33
Dihydroactinidiolide	0.4878	1.06	19.39
2-Hexenoic acid	0.4878	1.06	20.45
Pentadecanoic acid	0.4878	1.06	21.51
Hexanal	0.4878	1.06	22.57
2-Methoxy-3-isopropylpyrazine	0.4878	1.06	23.63
Hexanoic acid	0.4878	1.06	24.69
β-Linalool	0.4878	1.06	25.75
Lactic acid	0.4878	1.06	26.81
β-Sitosterol	0.4878	1.06	27.87

Linalool oxide	0.4878	1.06	28.93
Ethyl Benzoate	0.4242	0.9217	29.85
Isopropyl tetradecanoate	0.4242	0.9217	30.77
Hexanol	0.4242	0.9217	31.7
Tridecanoic acid	0.4242	0.9217	32.62
12-Methyltridecanoic acid	0.4242	0.9217	33.54
Nonanol	0.4242	0.9217	34.46
3-Hydroxy-2-methylpyran-4-one	0.4242	0.9217	35.38
Isododecanol	0.4242	0.9217	36.3
α-Ionona	0.4242	0.9217	37.23
2-Methoxybenzoic acid	0.4242	0.9217	38.15
Ethyl tetradecanoate	0.4242	0.9217	39.07
Heptanol	0.4242	0.9217	39.99
3-Ethoxy-4-hydroxybenzaldehyde	0.4242	0.9217	40.91
2-Methyl-4-pentenoic acid	0.4242	0.9217	41.83
Ethyl octadecanoate	0.4242	0.9217	42.76
Heptadecane	0.4242	0.9217	43.68
5,5-Dimethyl-3-oxo-1-cyclohexene-1-carboxaldehyde	0.4242	0.9217	44.6
4-Methylvaleric acid	0.4242	0.9217	45.52
1,2-Dimethoxybenzene	0.4242	0.9217	46.44
Heptanal	0.4242	0.9217	47.37
2-Isopropyl-3-metoxypirazina	0.4242	0.9217	48.29
1,2-Dihydroxy-4-methylpentane	0.4242	0.9217	49.21
3-(4-(tert-butyl)phenyl-2-methylpropanal	0.4242	0.9217	50.13
Benzyl salicylate	0.4242	0.9217	51.05
Octadecanal	0.4242	0.9217	51.97
Guaiacol	0.4242	0.9217	52.9
3-Ethyl-4-methyl-1H-pyrrole-2,5-dione	0.4242	0.9217	53.82
Nonadecane	0.4242	0.9217	54.74
Neophytadiene	0.4242	0.9217	55.66
Methyl 2-methoxy benzoate	0.4242	0.9217	56.58
1-Methyl-1(4-methyl-3-cyclohexenyl)ethanol	0.4242	0.9217	57.5
1-Tetradecanol	0.4242	0.9217	58.43
α-Isomethylionone	0.4242	0.9217	59.35
1-Octadecanol	0.4242	0.9217	60.27
Ethyl salycilate	0.4242	0.9217	61.19
Octanoic acid	0.4242	0.9217	62.11
α-Hexylcinnamaldehyde	0.4242	0.9217	63.04
Methyl benzoate	0.4242	0.9217	63.96
trans-Linalool oxide	0.4242	0.9217	64.88
(Z)-2-Hexen-1-ol	0.3781	0.8217	65.7
Octanal	0.3781	0.8217	66.52
Benzaldehyde	0.3781	0.8217	67.34
1-Octanol	0.3781	0.8217	68.17
Glutaric acid	0.3781	0.8217	68.99
15-Methylhexadecanoic acid	0.3781	0.8217	69.81
1,2-Dihydro-1,1,6-trimethyl naphthalene	0.3781	0.8217	70.63
4-Oxopentanoic acid	0.3781	0.8217	71.45

Nonanedioic acid	0.3781	0.8217	72.27
5-Hexen-2-one	0.3781	0.8217	73.1
Benzylmethylether	0.3781	0.8217	73.92
3-Methyl-2-pentenoic acid	0.3781	0.8217	74.74
Butanedioic acid	0.3781	0.8217	75.56
1,5,5-Trimethyl-3-methylene cyclohexene	0.3781	0.8217	76.38
10,18,Bisnorabieta-8,11,13.triene	0.3781	0.8217	77.2
1-(1-cyclohexen-1-yl)(-1-1-Butenone)	0.3781	0.8217	78.03
Gliceric acid	0.3781	0.8217	78.85
Methyl tetradecanoate	0.3781	0.8217	79.67
Limonene	0.3781	0.8217	80.49
p-Hydroxybenzoic acid	0.3781	0.8217	81.31
α-Terpineol	0.3781	0.8217	82.13
Methyl salicylate	0.3492	0.7587	82.89
Geraniol	0.3492	0.7587	83.65
2-Ethylhexanoic acid	0.3492	0.7587	84.41
2,5-Dimethoxy benzenemethanol acetate	0.3492	0.7587	85.17
2-(1-Hydroxybut-2-enylidene)cyclohexanone	0.3492	0.7587	85.93
2,2,6-Trimethylcyclohexanone	0.3492	0.7587	86.69
Eicosane	0.3492	0.7587	87.45
Heneicosane	0.3492	0.7587	88.2
Docosane	0.3492	0.7587	88.96
Methyl octadecanoate	0.3492	0.7587	89.72
3-Isobutyl-2-methoxypyrazine	0.3492	0.7587	90.48
28-Nor-17β(H)-hopane	0.3492	0.7587	91.24
Ethyl 2-(5-methyl-5-vinyltetrahydrofuran-2-yl)propan-2-yl carbonate	0.3492	0.7587	92
Isophorone	0.3348	0.7275	92.73
1-Hexadecanol	0.3348	0.7275	93.45
Benzophenone	0.3348	0.7275	94.18
Ethyl Dodecanoate	0.3348	0.7275	94.91
Benzeneacetic acid	0.3348	0.7275	95.64
Salicylic acid	0.3348	0.7275	96.36
2,3-Dihydro-2,2,4,6-tetramethylbenzofuran	0.3348	0.7275	97.09
1-Dodecanol	0.3348	0.7275	97.82
Decanoic acid	0.3348	0.7275	98.55
Octacosanol	0.3348	0.7275	99.27
(Z)-9-Octadecenoic acid	0.3348	0.7275	100
p-Vinylguaiacol	3.04E-09	6.60E-09	100
Dehydroabietic acid	2.16E-09	4.69E-09	100
Dodecanoic acid	9.91E-10	2.15E-09	100
Hexadecanoic acid	8.76E-10	1.90E-09	100
Tetradecanoic acid	4.79E-10	1.04E-09	100
Octadecanoic acid	3.99E-10	8.68E-10	100
2-Ethylhexanol	3.82E-10	8.30E-10	100
Benzoic acid	3.65E-10	7.93E-10	100
4-Hydroxy-3-methoxybenzaldehyde	3.34E-10	7.25E-10	100
Benzylalcohol	3.12E-10	6.79E-10	100

(Z,Z)-9,12-Octadecadienoic acid	2.73E-10	5.93E-10	100
Nonanal	2.21E-10	4.79E-10	100
Nonanoic acid	1.89E-10	4.11E-10	100
Phenylacetone	6.50E-11	1.41E-10	100
Heptanoic acid	5.70E-11	1.24E-10	100
Phenylacetaldehyde	5.66E-11	1.23E-10	100
Undecanoic acid	1.46E-11	3.17E-11	100

Table S4. SIMPER analysis of *Opuntia ficus-indica* (OFI) Esmeralda and OFI Esmeralda infested by *Dactylopius coccus*.

Compounds	Averange dissimilarity	Contribution %	Cumulative %
Isopropyl tetradecanoate	0.9434	1.389	1.389
Ethyl Benzoate	0.9434	1.389	2.778
Tridecanoic acid	0.9434	1.389	4.167
Hexanol	0.9434	1.389	5.556
Nonanol	0.9434	1.389	6.944
3-Hydroxy-2-methylpyran-4-one	0.9434	1.389	8.333
12-Methyltridecanoic acid	0.9434	1.389	9.722
α-Ionona	0.9434	1.389	11.11
Isododecanol	0.9434	1.389	12.5
2-Methoxybenzoic acid	0.9434	1.389	13.89
Ethyl tetradecanoate	0.9434	1.389	15.28
3-Ethoxy-4-hydroxybenzaldehyde	0.9434	1.389	16.67
2-Methyl-4-pentenoic acid	0.9434	1.389	18.06
Heptanol	0.9434	1.389	19.44
Ethyl octadecanoate	0.9434	1.389	20.83
Heptadecane	0.9434	1.389	22.22
4-Oxoisophorone	0.9434	1.389	23.61
Decanal	0.9434	1.389	25
4-Methylvaleric acid	0.9434	1.389	26.39
Benzaldehyde	0.9434	1.389	27.78
5,5-Dimethyl-3-oxo-1-cyclohexene-1-carboxaldehyde	0.9434	1.389	29.17
Glutaric acid	0.9434	1.389	30.56
1,2-Dimethoxybenzene	0.9434	1.389	31.94
15-Methylhexadecanoic acid	0.9434	1.389	33.33
Octanal	0.9434	1.389	34.72
1,2-Dihydro-1,1,6-trimethyl naphthalene	0.9434	1.389	36.11
β-Damascenone	0.9434	1.389	37.5
Heptanal	0.9434	1.389	38.89
Linalool	0.9434	1.389	40.28
4-Oxopentanoic acid	0.9434	1.389	41.67
2-Isopropyl-3-metoxypirazina	0.9434	1.389	43.06
1,2-Dihydroxy-4-methylpentane	0.9434	1.389	44.44
Nonanedioic acid	0.9434	1.389	45.83
1-Octanol	0.9434	1.389	47.22
5-Hexen-2-one	0.9434	1.389	48.61

3-(4-(tert-butyl)phenyl-2-methylpropanal	0.9434	1.389	50
Benzyl salicylate	0.9434	1.389	51.39
Benzylmethylether	0.9434	1.389	52.78
Octadecanal	0.9434	1.389	54.17
Glycerol	0.9434	1.389	55.56
Nerol	0.9434	1.389	56.94
Guaiacol	0.9434	1.389	58.33
3-Ethyl-4-methyl-1H-pyrrole-2,5-dione	0.9434	1.389	59.72
3-Methyl-2-pentenoic acid	0.9434	1.389	61.11
(Z)-2-Hexen-1-ol	0.9434	1.389	62.5
Nonadecane	0.9434	1.389	63.89
Neophytadiene	0.9434	1.389	65.28
β-Ionone	0.9434	1.389	66.67
Methyl benzoate	0.9434	1.389	68.06
Methyl 2-methoxy benzoate	0.9434	1.389	69.44
Butanedioic acid	0.9434	1.389	70.83
1,5,5-Trimethyl-3-methylene cyclohexene	0.9434	1.389	72.22
Acetophenone	0.9434	1.389	73.61
10,18,Bisnorabieta-8,11,13.triene	0.9434	1.389	75
1-Methyl-1(4-methyl-3-cyclohexenyl)ethanol	0.9434	1.389	76.39
1-Tetradecanol	0.9434	1.389	77.78
1-(1-cyclohexen-1-yl)(-1-1-Butenone)	0.9434	1.389	79.17
Gliceric acid	0.9434	1.389	80.56
Methyl tetradecanoate	0.9434	1.389	81.94
Limonene	0.9434	1.389	83.33
2-Nonenoic acid	0.9434	1.389	84.72
α-Isomethylionone	0.9434	1.389	86.11
1-Octadecanol	0.9434	1.389	87.5
p-Hydroxybenzoic acid	0.9434	1.389	88.89
Ethyl salycilate	0.9434	1.389	90.28
α-Hexylcinnamaldehyde	0.9434	1.389	91.67
Octanoic acid	0.9434	1.389	93.06
Methyl benzoate	0.9434	1.389	94.44
3,7,11,15-Tetramethyl-2-hexadecenol	0.9434	1.389	95.83
α-Terpineol	0.9434	1.389	97.22
Benzyl Benzoate	0.9434	1.389	98.61
trans-Linalool oxide	0.9434	1.389	100
p-Vinylguaiacol	3.89E-09	5.73E-09	100
Methyl salicylate	2.53E-09	3.72E-09	100
β-Linalool	2.08E-09	3.07E-09	100
Linalool oxide	1.50E-09	2.21E-09	100
Dodecanoic acid	1.29E-09	1.90E-09	100
Hexadecanoic acid	1.02E-09	1.49E-09	100
Tetradecanoic acid	7.12E-10	1.05E-09	100
Lactic acid	6.99E-10	1.03E-09	100
Benzoic acid	5.27E-10	7.76E-10	100
(Z,Z)-9,12-Octadecadienoic acid	3.82E-10	5.63E-10	100
Octadecanoic acid	3.38E-10	4.98E-10	100
1-Hexadecanol	3.30E-10	4.85E-10	100
Dehydroabietic acid	2.94E-10	4.33E-10	100

Hexadecane	2.72E-10	4.01E-10	100
4-Hydroxy-3-methoxybenzaldehyde	2.28E-10	3.36E-10	100
Nonanal	2.20E-10	3.23E-10	100
(Z)-9-Octadecenoic acid	1.80E-10	2.65E-10	100
2-Ethylhexanol	1.58E-10	2.33E-10	100
Nonanoic acid	1.45E-10	2.13E-10	100
2-Hexenoic acid	1.32E-10	1.94E-10	100
Benzylalcohol	1.27E-10	1.88E-10	100
Isophorone	1.01E-10	1.49E-10	100
Diethyl acetal hexanal	9.23E-11	1.36E-10	100
Hexylsalicylate	9.23E-11	1.36E-10	100
Heptanoic acid	8.79E-11	1.29E-10	100
Nonanaldimethylacetal	7.03E-11	1.04E-10	100
Decanoic acid	6.15E-11	9.06E-11	100
1-Dodecanol	5.71E-11	8.41E-11	100
Geraniol	4.83E-11	7.11E-11	100
Ethyl Dodecanoate	3.95E-11	5.82E-11	100
Dihydroactinidiolide	3.51E-11	5.17E-11	100
Phenol	2.64E-11	3.88E-11	100
Phenylacetaldehyde	2.64E-11	3.88E-11	100
Phenylacetone	1.76E-11	2.59E-11	100
Benzophenone	1.32E-11	1.94E-11	100
Hexanal	1.32E-11	1.94E-11	100
Undecanoic acid	8.79E-12	1.29E-11	100
Pentadecanoic acid	4.39E-12	6.47E-12	100
Hexanoic acid	4.39E-12	6.47E-12	100
Heneicosane	0	0	100
Eicosane	0	0	100
Octacosanol	0	0	100
Salicylic acid	0	0	100
2,5-Dimethoxy benzenemethanol acetate	0	0	100
Benzeneacetic acid	0	0	100
β-Sitosterol	0	0	100
28-Nor-17β(H)-hopane	0	0	100
Methyl octadecanoate	0	0	100
2-Ethylhexanoic acid	0	0	100
2-(1-Hydroxybut-2-enylidene)cyclohexanone	0	0	100
2,2,6-Trimethylcyclohexanone	0	0	100
2,3-Dihydro-2,2,4,6-tetramethylbenzofuran	0	0	100
3-Isobutyl-2-methoxypyrazine	0	0	100
Ethyl 2-(5-methyl-5-vinyltetrahydrofuran-2-yl)propan-2-yl carbonate	0	0	100
2-Methoxy-3-isopropylpyrazine	0	0	100
Docosane	0	0	100

Table S5. SIMPER analysis of OFI Rojo Pelón and OFI Rojo Pelón infested by *Dactylopius opuntiae*

Compounds	Averange dissimilarity	Contribution %	Cumulative %
4-Oxoisophorone	0.5882	2.778	2.778
2-Ethylhexanoic acid	0.5882	2.778	5.556
2,5-Dimethoxy benzenemethanol acetate	0.5882	2.778	8.333
2-(1-Hydroxybut-2-enylidene)cyclohexanone	0.5882	2.778	11.11
Decanal	0.5882	2.778	13.89
2,2,6-Trimethylcyclohexanone	0.5882	2.778	16.67
Benzyl Benzoate	0.5882	2.778	19.44
Benzeneacetic acid	0.5882	2.778	22.22
Isophorone	0.5882	2.778	25
Salicylic acid	0.5882	2.778	27.78
β-Ionone	0.5882	2.778	30.56
Eicosane	0.5882	2.778	33.33
Heneicosane	0.5882	2.778	36.11
Docosane	0.5882	2.778	38.89
Methyl salicylate	0.5882	2.778	41.67
Methyl octadecanoate	0.5882	2.778	44.44
Ethyl Dodecanoate	0.5882	2.778	47.22
2-Nonenoic acid	0.5882	2.778	50
2,3-Dihydro-2,2,4,6-tetramethylbenzofuran	0.5882	2.778	52.78
Benzophenone	0.5882	2.778	55.56
3-Isobutyl-2-methoxypyrazine	0.5882	2.778	58.33
Acetophenone	0.5882	2.778	61.11
28-Nor-17β(H)-hopane	0.5882	2.778	63.89
1-Hexadecanol	0.5882	2.778	66.67
β-Damascenone	0.5882	2.778	69.44
1-Dodecanol	0.5882	2.778	72.22
Nerol	0.5882	2.778	75
Decanoic acid	0.5882	2.778	77.78
Methyl benzoate	0.5882	2.778	80.56
Octacosanol	0.5882	2.778	83.33
Glycerol	0.5882	2.778	86.11
Geraniol	0.5882	2.778	88.89
(Z)-9-Octadecenoic acid	0.5882	2.778	91.67
3,7,11,15-Tetramethyl-2-hexadecenol	0.5882	2.778	94.44
Linalool	0.5882	2.778	97.22
Ethyl 2-(5-methyl-5-vinyltetrahydrofuran-2-yl)propan-2-yl carbonate	0.5882	2.778	100
β-Sitosterol	1.70E-09	8.02E-09	100
Dodecanoic acid	1.33E-09	6.26E-09	100
(Z)-2-Hexen-1-ol	9.42E-10	4.45E-09	100
p-Vinylguaiacol	6.46E-10	3.05E-09	100
Octadecanoic acid	5.94E-10	2.81E-09	100
Benzoic acid	4.27E-10	2.02E-09	100
Tetradecanoic acid	3.75E-10	1.77E-09	100

(Z,Z)-9,12-Octadecadienoic acid	3.62E-10	1.71E-09	100
Benzylalcohol	3.23E-10	1.53E-09	100
4-Hydroxy-3-methoxybenzaldehyde	3.18E-10	1.50E-09	100
Hexadecanoic acid	3.01E-10	1.42E-09	100
2-Methoxy-3-isopropylpyrazine	2.05E-10	9.70E-10	100
2-Ethylhexanol	1.75E-10	8.28E-10	100
Nonanal	1.12E-10	5.30E-10	100
Octanal	8.49E-11	4.01E-10	100
Nonanoic acid	7.40E-11	3.49E-10	100
1-Octanol	6.57E-11	3.10E-10	100
Benzaldehyde	6.30E-11	2.98E-10	100
Ethyl Benzoate	4.38E-11	2.07E-10	100
Heptanoic acid	3.84E-11	1.81E-10	100
Octanoic acid	3.56E-11	1.68E-10	100
Phenylacetone	2.19E-11	1.04E-10	100
Dehydroabietic acid	1.92E-11	9.06E-11	100
Undecanoic acid	1.10E-11	5.17E-11	100
Phenylacetaldehyde	2.74E-12	1.29E-11	100
3-Ethoxy-4-hydroxybenzaldehyde	0	0	100
Heptadecane	0	0	100
Hexylsalicylate	0	0	100
3-(4-(tert-butyl)phenyl-2-methylpropanal	0	0	100
1,2-Dihydro-1,1,6-trimethyl naphthalene	0	0	100
Nonanaldimethylacetal	0	0	100
p-Hydroxybenzoic acid	0	0	100
1-Octadecanol	0	0	100
1-Tetradecanol	0	0	100
Isododecanol	0	0	100
1-Methyl-1(4-methyl-3-cyclohexenyl)ethanol	0	0	100
4-Methylvaleric acid	0	0	100
5,5-Dimethyl-3-oxo-1-cyclohexene-1-carboxaldehyde	0	0	100
Guaiacol	0	0	100
Diethyl acetal hexanal	0	0	100
Heptanol	0	0	100
Phenol	0	0	100
Heptanal	0	0	100
Hexanal	0	0	100
1,2-Dihydroxy-4-methylpentane	0	0	100
Glutaric acid	0	0	100
Neophytadiene	0	0	100
Ethyl octadecanoate	0	0	100
Dihydroactinidiolide	0	0	100
Methyl 2-methoxy benzoate	0	0	100
2-Hexenoic acid	0	0	100
α-Ionona	0	0	100
2-Methoxybenzoic acid	0	0	100
α-Terpineol	0	0	100
β-Linalool	0	0	100

15-Methylhexadecanoic acid	0	0	100
Gliceric acid	0	0	100
α-Isomethylionone	0	0	100
1,5,5-Trimethyl-3-methylene cyclohexene	0	0	100
trans-Linalool oxide	0	0	100
Linalool oxide	0	0	100
Limonene	0	0	100
Benzyl salicylate	0	0	100
Butanedioic acid	0	0	100
Hexanol	0	0	100
1-(1-cyclohexen-1-yl)(-1-1-Butenone)	0	0	100
Isopropyl tetradecanoate	0	0	100
Pentadecanoic acid	0	0	100
Nonanedioic acid	0	0	100
Ethyl tetradecanoate	0	0	100
Ethyl salycilate	0	0	100
5-Hexen-2-one	0	0	100
Nonanol	0	0	100
1,2-Dimethoxybenzene	0	0	100
Benzylmethylether	0	0	100
Methyl benzoate	0	0	100
Lactic acid	0	0	100
3-Hydroxy-2-methylpyran-4-one	0	0	100
3-Ethyl-4-methyl-1H-pyrrole-2,5-dione	0	0	100
12-Methyltridecanoic acid	0	0	100
2-Isopropyl-3-metoxypirazina	0	0	100
Tridecanoic acid	0	0	100
Octadecanal	0	0	100
α-Hexylcinnamaldehyde	0	0	100
10,18,Bisnorabieta-8,11,13.triene	0	0	100
Hexadecane	0	0	100
Methyl tetradecanoate	0	0	100
Nonadecane	0	0	100
2-Methyl-4-pentenoic acid	0	0	100
4-Oxopentanoic acid	0	0	100
3-Methyl-2-pentenoic acid	0	0	100
		0	
Hexanoic acid	0		100

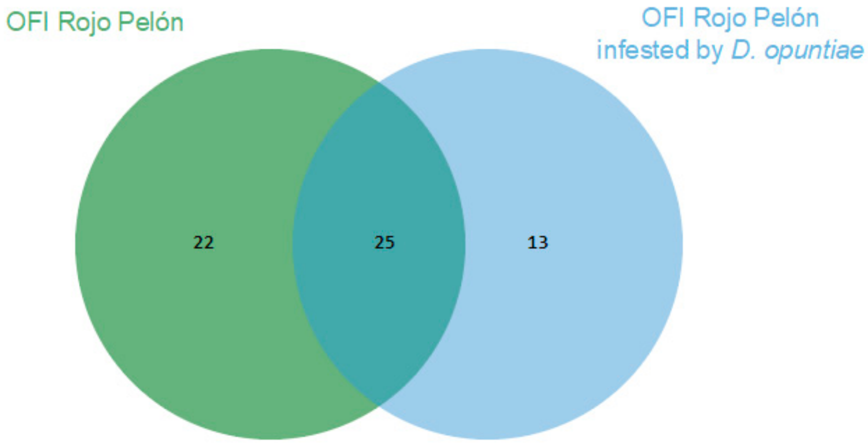

Figure S1. Comparison of volatilomes of the *Opuntia ficus-indica* (OFI) Rojo Pelón uninfested and infested by *D. opuntiae* using Venn diagram.

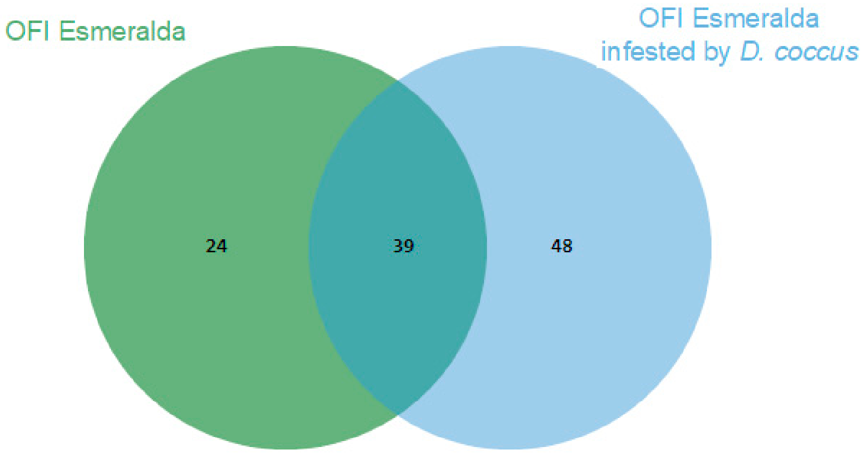

Figure S2. Comparison of volatilomes of the *Opuntia ficus-indica* (OFI) Esmeralda uninfested and infested by *D. coccus* using Venn diagram.

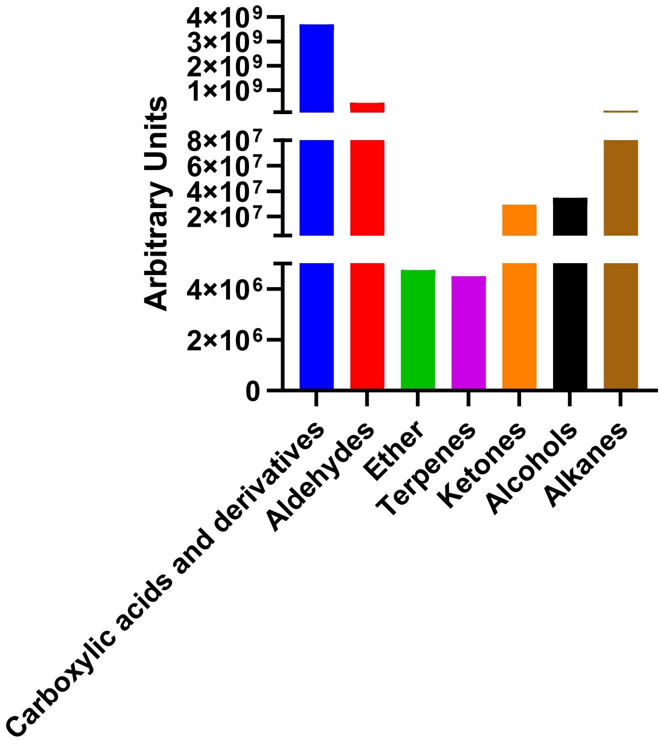

Figure S3. *Dactylopius opuntiae* compounds grouped. The data are presented as the mean of the peak area of each compound (grouped by type) and the range of the data.

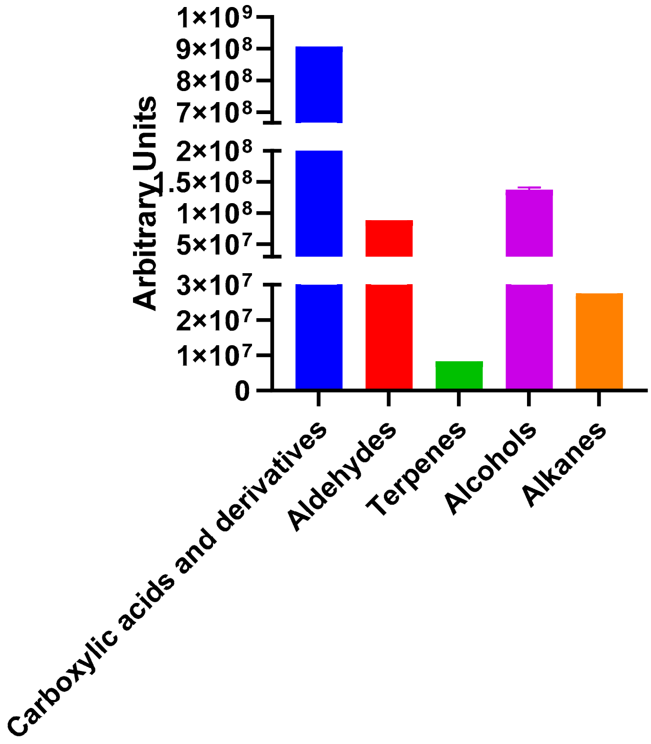

Figure S4. *Dactylopius coccus* compounds grouped. The data are presented as the mean of the peak area of each compound (grouped by type) and the range of the data.

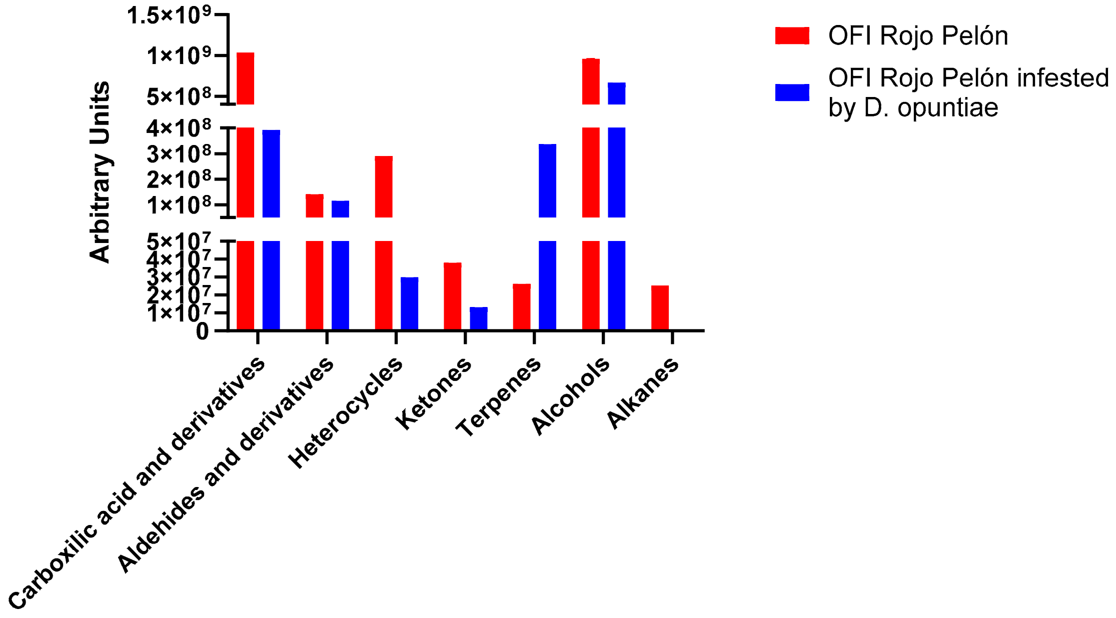

Figure S5. OFI Rojo Pelón compounds grouped. The data are presented as the mean of the peak area of each compound (grouped by type) and the range of the data.

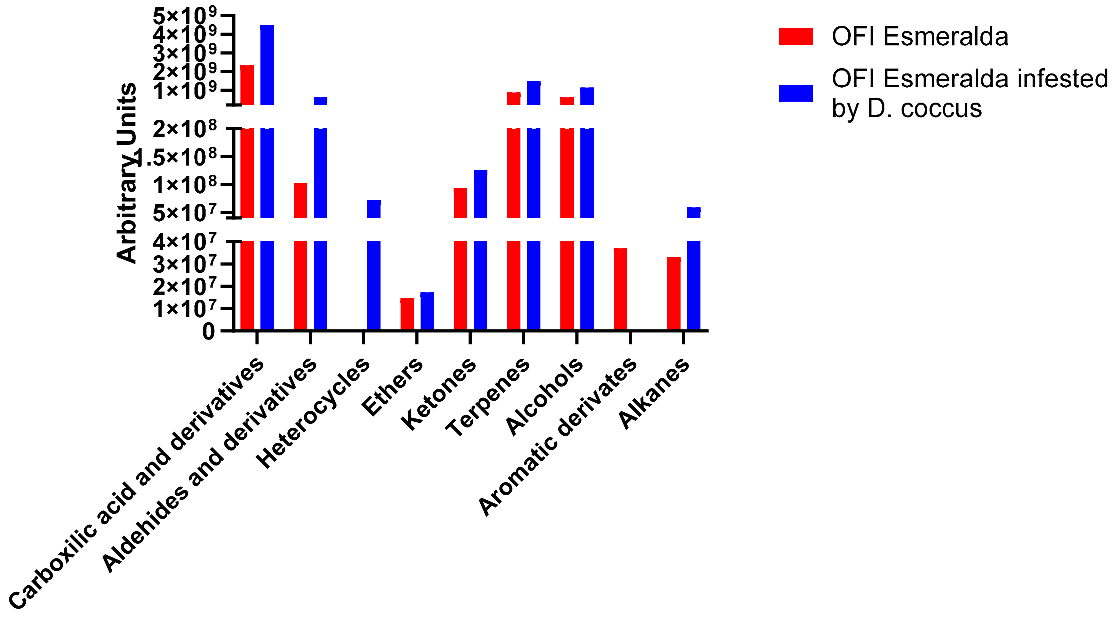

Figure S6. OFI Esmeralda compounds grouped. The data are presented as the mean of the peak area of each compound (grouped by type) and the range of the data.
